# Dermoscopy of eccrine syringofibroadenoma: A rare cutaneous adnexal tumor

**DOI:** 10.1016/j.jdcr.2026.06.001

**Published:** 2026-06-08

**Authors:** Jessica Reyes, Yosbeli Ramírez, Federico Fuentes, Karla Osorio, Sofia Moran, Ludwin Castro

**Affiliations:** a Dermatology Resident, Department of Dermatology, Hospital Nacional Zacamil El Salvador, San Salvador, El Salvador; b Dermatologist, Department of Dermatology, Hospital Nacional Zacamil, San Salvador, El Salvador

**Keywords:** acrosyringeal nevus, dermoscopy, eccrine syringofibroadenoma, pathology

## Clinical presentation

**Fig 1:**
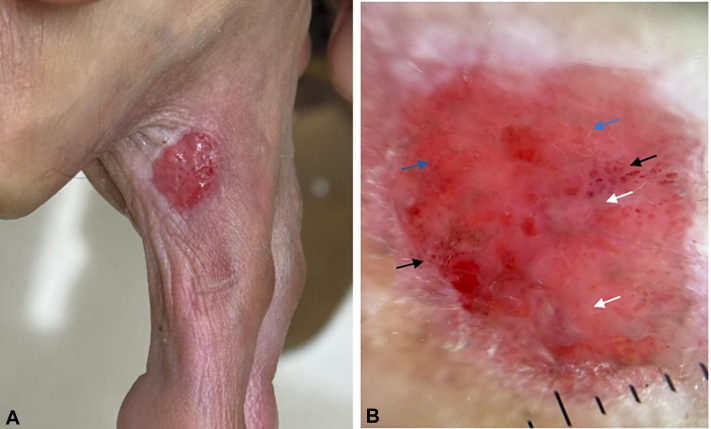
**A,** Clinical presentation of eccrine syringofibroadenoma on the dorsum of the left foot. **B,** Dermatoscopy: glomerular vessels (*black arrows*) and white structureless areas (*white arrows*) on pink structureless areas (*blue arrows*) were observed on the dorsum of the foot.

A 49-year-old male patient with a 6-month history of a progressively enlarging, asymptomatic, erythematous lesion measuring of 2.4 × 1.6 cm on the first interdigital space of the right foot (Fig 1, *A*). There was no history of preceding local trauma.

## Dermatoscopic appearance

Dermoscopy (DL200 Hybrid dermatoscope, DermLite; 20× magnification) revealed glomerular vessels (black arrows), white structureless areas (asterisk), and pink structureless areas (white arrows) on the dorsum of the foot (Fig 1, *B*).

## Histologic diagnosis

**Fig 2:**
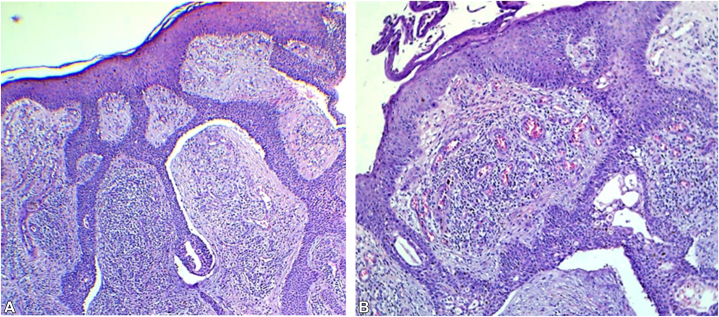
**A,** H&E showing thin anastomosing strands of small epithelial cells extending from the epidermis, corresponding dermoscopically to pink structureless areas. **B,** Fibrotic stroma and ductal structures containing eosinophilic cuticular material corresponding to white structureless areas along vascular proliferation corresponding to glomerular vessels.^3^*H&E*, Hematoxylin and eosin.

The dermatopathologic examination demonstrated irregular acanthosis with thin, anastomosing strands of epithelial cells enclosing a fibrovascular stroma (Fig 2, *A*). Ductal structures containing eosinophilic cuticular material (Fig 2, *B*).

Key message

Eccrine syringofibroadenoma (also referred to as acrosyringeal nevus) is a rare adnexal neoplasm with eccrine differentiation.^1^ Dermoscopy may provide valuable clues for its early recognition, facilitating clinical suspicion before histopathologic confirmation. Clinically, the presentation of eccrine syringofibroadenoma is variable and may manifest as a solitary lesion or as multiple papules or nodules that may be distributed in a symmetrical or linear, nevus-like pattern.^1^ It is categorized into 5 clinical subtypes: solitary eccrine syringofibroadenoma, multiple lesions associated with ectodermal dysplasia, multiple lesions without accompanying skin abnormalities, nonfamilial unilateral linear lesions, and familial linear cases.^2^ Although recurrence is uncommon, malignant transformation has been reported in long-standing cases.^2^

## Conflicts of interest

None disclosed.
